# New Horizons: The Evolution of Nuclear Medicine in the Diagnosis and Treatment of Pancreatic Neuroendocrine Tumors—A Case Report

**DOI:** 10.3390/jcm14134432

**Published:** 2025-06-22

**Authors:** Annamária Bakos, László Libor, Béla Vasas, Kristóf Apró, Gábor Sipka, László Pávics, Zsuzsanna Valkusz, Anikó Maráz, Zsuzsanna Besenyi

**Affiliations:** 1 Department of Nuclear Medicine, University of Szeged, 6720 Szeged, Hungary; apro.kristof@med.u-szeged.hu (K.A.); sipka.gabor@med.u-szeged.hu (G.S.); pavics.laszlo@med.u-szeged.hu (L.P.); besenyi.zsuzsanna@med.u-szeged.hu (Z.B.); 2 Department of Surgery, University of Szeged, 6720 Szeged, Hungary; liborlasz@gmail.com; 3 Department of Pathology, University of Szeged, 6720 Szeged, Hungary; vasas.bela@med.u-szeged.hu; 4 Department of Internal Medicine, University of Szeged, 6720 Szeged, Hungary; valkusz.zsuzsanna@med.u-szeged.hu; 5 Department of Oncotherapy, University of Szeged, 6720 Szeged, Hungary; maraz.aniko@med.u-szeged.hu

**Keywords:** pancreatic neuroendocrine tumor, clinical challenges, personalized oncology functional volumetry, peptide receptor radionuclide therapy, hepatectomy, somatostatin receptor scintigraphy

## Abstract

**Background**: Pancreatic neuroendocrine tumors (PanNETs) are relatively rare neoplasms with heterogeneous behavior, ranging from indolent to aggressive disease. The evolution of nuclear medicine has allowed the development of an efficient and advanced toolkit for the diagnosis and treatment of PanNETs. **Case**: A 45-year-old woman was diagnosed with a grade 1 PanNET and multiple liver metastases. She underwent distal pancreatectomy with splenectomy, extended liver resection, and radiofrequency ablation (RFA). Surgical planning was guided by [^99m^Tc]Tc-EDDA/HYNIC-TOC SPECT/CT (single-photon emission computed tomography/computed tomography) and preoperative [^99m^Tc]Tc-mebrofenin-based functional liver volumetry. Functional liver volumetry based on dynamic [^99m^Tc]Tc-mebrofenin SPECT/CT facilitated precise surgical planning and reliable assessment of the efficacy of parenchymal modulation, thereby aiding in the prevention of post-hepatectomy liver failure. Liver fibrosis was non-invasively evaluated using two-dimensional shear wave elastography (2D-SWE). Tumor progression was monitored using somatostatin receptor scintigraphy, chromogranin A, and contrast-enhanced CT. Recurrent disease was treated with somatostatin analogues (SSAs) and [^177^Lu]Lu-DOTA-TATE peptide receptor radionuclide therapy (PRRT). Despite progression to grade 3 disease (Ki-67 from 1% to 30%), the patient remains alive 53 months post-diagnosis, in complete remission, with an ECOG (Eastern Cooperative Oncology Group) status of 0. **Conclusions**: Functional imaging played a pivotal role in guiding therapeutic decisions throughout the disease course. This case not only underscores the clinical utility of advanced nuclear imaging but also illustrates the dynamic nature of pancreatic neuroendocrine tumors. The transition from low-grade to high-grade disease highlights the need for further studies on tumor progression mechanisms and the potential role of adjuvant therapies in managing PanNETs.

## 1. Introduction

Neuroendocrine neoplasms (NENs) originate from the diffuse neuroendocrine cell system and can develop in various locations throughout the body. Gastroenteropancreatic neuroendocrine tumors (GEP-NETs) represent a heterogeneous and rare group of neoplasms. Their incidence has increased in recent decades, largely due to advancements in imaging techniques. In Europe, GEP-NETs are now estimated to occur in 1.33 to 2.33 cases per 100,000 individuals [1,2,3].

Pancreatic neuroendocrine tumors (PanNETs) are relatively rare, accounting for approximately 2% of all pancreatic tumors [4,5]. While histological differentiation of neuroendocrine tumors (NETs) does not always predict their clinical behavior, even small, low-grade (grade 1 or 2) PanNETs can spread to the liver or lymph nodes [6].

According to the European Society of Medical Oncology (ESMO) guidelines, surgical intervention is the preferred treatment for resectable PanNETs [5]. Curative resection (R0 or R1) is associated with a 5-year overall survival (OS) rate of approximately 85% [7]. In some cases, combining surgical resection with radiofrequency ablation (RFA) can enable the complete removal of tumors in areas inaccessible by surgery alone [5]. However, in cases requiring major liver resection, meticulous attention is essential to ensure patient safety. Post-hepatectomy liver failure (PHLF) remains a significant cause of severe morbidity, with reported incidence rates ranging from 9% to 30% [8]. Preoperative assessment of liver function is crucial for identifying patients at increased risk of post-hepatectomy liver failure (PHLF). The E-AHPBA–ESSO–ESSR Innsbruck consensus guidelines recommend evaluating both global liver function and the volumetric and functional fraction of the future liver remnant (FLR) prior to surgery. Several clinical scoring systems are used to assess global liver function, including the MELD-Na score (Model for End-Stage Liver Disease), the APRI score (AST to Platelet Ratio Index), the CTP score (Child–Turcotte–Pugh score), and the ICG (indocyanine green) clearance test. CT (computed tomography) volumetry is commonly employed to determine the volumetric FLR fraction. Additionally, two-dimensional ultrasound shear wave elastography (2D-SWE) provides a non-invasive method for evaluating liver stiffness and assessing the degree of fibrosis [9]. [^99m^Tc]Tc-mebrofenin SPECT/CT offers the advantage of providing simultaneous volumetric and quantitative functional information. However, its use remains limited to a small number of specialized centers [8].

Currently, no data in the literature supports the use of adjuvant therapy for grade 1 (G1) or grade 2 (G2) neuroendocrine tumors (NETs), as prospective clinical studies are lacking. Medical therapy is recommended based on established guidelines to manage tumor-associated symptoms and control tumor growth. Somatostatin analogues (SSAs) serve as antiproliferative agents in metastatic PanNETs. In cases of progressive disease, peptide receptor radionuclide therapy (PRRT) with [^177^Lu]Lu-DOTA-TATE may be considered, as outlined by the ESMO guidelines, to improve symptoms. PRRT is specifically indicated for progressive somatostatin receptor (SSTR)-positive NETs with homogenous SSTR expression, confirmed through SSTR imaging [5].

In this case report, we present the multidisciplinary management of a PanNET, utilizing some less commonly applied methods in clinical practice.

## 2. The Case

### 2.1. Patient Presentation and Initial Diagnosis

In autumn 2020, a 45-year-old female patient underwent evaluation for nonspecific abdominal complaints. An abdominal ultrasound revealed multiple hepatic nodules and a lesion in the pancreatic tail. On September 16, 2020, an abdominal CT scan confirmed the presence of a mass in the pancreatic tail along with multiple liver metastases. Endoscopic ultrasound-guided fine-needle aspiration (EUS-FNA) was performed on the pancreatic tail lesion, with additional biopsies taken from a peripancreatic lymph node and liver metastases. Histopathological analysis confirmed a grade 1 neuroendocrine tumor of pancreatic origin, with a Ki-67 proliferation index of 1%. The patient had no prior personal or family history of tumor-related illnesses.

**Figure 1:**
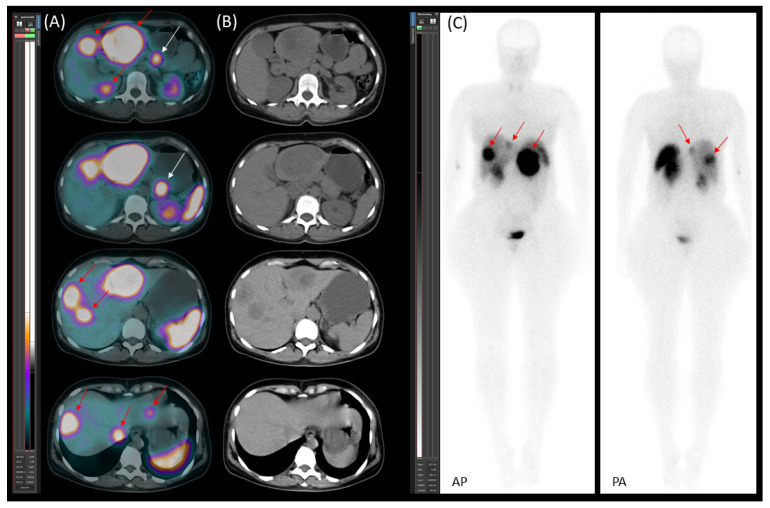
[^99m^Tc]Tc-EDDA/HYNIC-TOC scintigraphy with SPECT/CT. (**A**) Transaxial SPECT/CT, (**B**) transaxial CT, and (**C**) planar images (AP—anteroposterior; PA—posteroanterior) demonstrate somatostatin receptor (SSTR)-positive lesions. Multiple metastatic liver lesions are visible in all modalities (red arrows). The primary lesion in the pancreatic tail is clearly identified on the SPECT/CT image and is marked with a white arrow.

On 30 November 2020, [^99m^Tc]Tc-EDDA/HYNIC-TOC somatostatin receptor scintigraphy with SPECT/CT was performed using a native, low-dose protocol. Oral contrast (1000 mL of polyethylene glycol solution) was administered 60 min prior to imaging to promote bowel distention without introducing contrast material-induced attenuation correction artefacts. The imaging revealed increased radiopharmaceutical uptake in CT-identified lesions, consistent with somatostatin receptor expression (Figure 1). Serum chromogranin A (CgA) levels were moderately elevated at 108 ng/mL (reference range: 19.0–98.0).

A multidisciplinary team recommended surgical treatment. In preparation, the patient was started on somatostatin long-acting analogue therapy (lanreotide monotherapy, 120 mg intramuscularly).

### 2.2. Preoperative Planning and First-Stage Surgery

The surgical plan included resection of the pancreatic tail with splenectomy, right hemi-hepatectomy, and additional left hepatic metastasectomy to address metastases in both liver lobes. Since an extended liver resection was planned, meticulous preoperative planning was carried out.

As part of preoperative planning, the patient’s global liver function was first assessed using standard clinical scoring systems. The Model for End-Stage Liver Disease (MELD) score was 6, the aspartate aminotransferase (AST)-to-platelet ratio index (APRI) was 0.3, and the Child–Turcotte–Pugh (CTP) score was 5, all indicating normal liver function. Ultrasound 2D shear wave elastography was used to assess liver parenchyma in both lobes, revealing no signs of fibrosis (left lobe velocity: 1.3 m/s, right lobe velocity: 1.37 m/s).

Subsequently, functional volumetric analysis was performed using dynamic [^99m^Tc]Tc-mebrofenin SPECT/CT, a hepatocyte-specific radiotracer, to quantify the volumetric and functional proportion of the future liver remnant (FLR), as defined according to the surgical plan, relative to the total liver volume. This evaluation was essential to determine the feasibility and safety of proceeding with the planned surgical intervention [10]. Dynamic SPECT/CT (native, low-dose) imaging was performed using a three-headed integrated system equipped with a low-energy, high-resolution parallel-hole collimator (Mediso AnyScan TRIO, Mediso Medical Imaging Systems Ltd., Budapest, Hungary). Following intravenous administration of 300 MBq of the hepatocyte-specific radiotracer [^99m^Tc]Tc-mebrofenin, continuous dynamic SPECT acquisition was initiated. The patient was positioned supine, with the field of view (FOV) covering the liver and heart regions, including the liver parenchyma, cardiac structures, and the biliary system up to the choledochus. Dynamic imaging commenced upon visualization of tracer inflow into the aorta, capturing the time-dependent distribution of the radiotracer, including hepatic uptake (tracer extraction into hepatocytes), accumulation, and biliary excretion phases. Time–activity curves (TACs) were generated from the continuously acquired data using volume-of-interest (VOI) analysis based on the reconstructed SPECT images [10,11].

**Figure 2:**
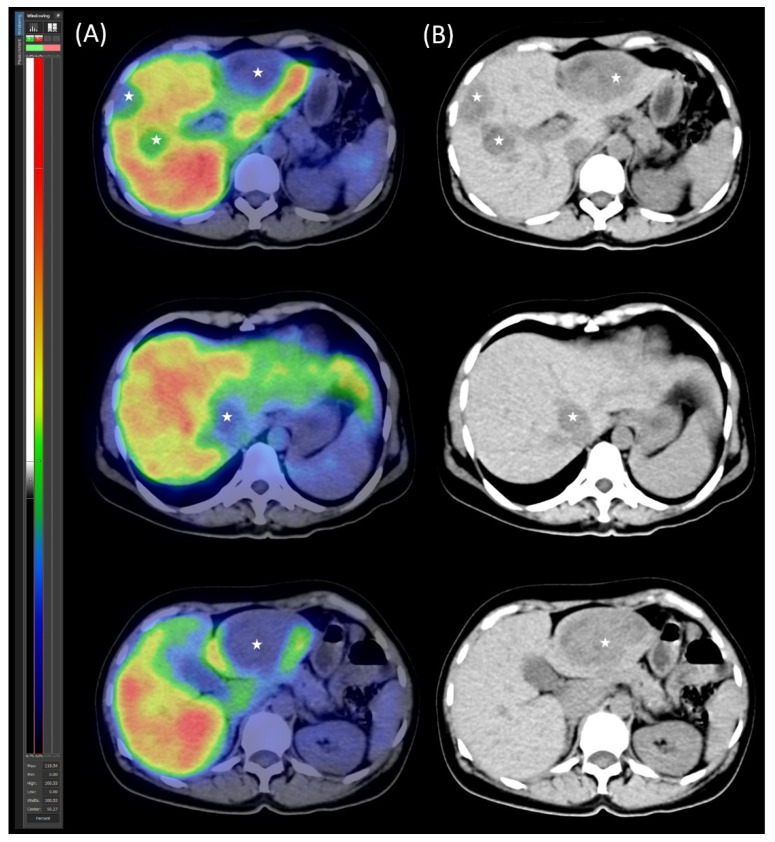
[^99m^Tc]Tc-mebrofenin SPECT/CT. (**A**) Transaxial SPECT/CT and (**B**) native low-dose CT images illustrate the distribution of functional hepatocytes and metastases. The metastases appear as hypodense, photopenic areas (marked with stars).

During the early, purely parenchymal phase of the SPECT scans (Figure 2)—prior to the appearance of biliary excretion—the functional volume rate of the future liver remnant (FLR-FV%) was determined. This was calculated as the ratio of the summed voxel counts within the future liver remnant (FLR-C) to the summed voxel counts of the total liver (TL-C), expressed as a percentage by multiplying the result by 100. No biliary tracer activity was observed on the SPECT images during this phase. Additionally, the volumetric fraction of the future liver remnant (FLR-V%) was calculated as the ratio of the FLR volume (FLR-V) to the tumor-free total liver volume (TL-V), also expressed as a percentage after multiplication by 100. These functional and volumetric assessments of the FLR were essential for preoperative evaluation, as estimation of both the volumetric and functional proportion of the future liver remnant, according to surgical recommendations, is critical for determining the feasibility and safety of a planned liver resection [8,10,12].

In order to evaluate the functional capacity of the hepatocytes within the future liver remnant, FLR filtration was also calculated, providing more accurate and quantitative data to facilitate surgical decision-making. Future liver remnant filtration (FLR-F, %/min) was calculated from the time–activity curves, using data acquired between 150 and 350 s post-injection, based on Ekman’s established formula [13]. The FLR filtration value was normalized to body surface area (BSA, %/min/m^2^) to account for individual metabolic differences, with BSA calculated using the Mosteller formula [14]. This normalization allowed for comparison of the obtained values with previously established cutoff thresholds in order to determine whether the FLR filtration was sufficient to safely proceed with surgery [11].

The analysis demonstrated a future liver remnant (FLR) volume fraction of 38% and a functional fraction of 29%, which, in the context of normal global liver function, was considered sufficient. However, the FLR filtration was 2.29%/min/m^2^, falling below the literature-based safety cutoff [10,11].

The following strategy was planned for liver resection: a two-stage hepatectomy combining metastasectomy and radiofrequency ablation (RFA) in the left lobe (FLR). Since removal of the metastasis located deep within the left lobe would have compromised the blood supply of segment II, ablation of this lesion was planned instead. Furthermore, as the surgical plan involved metastasectomy and RFA within the FLR, additional parenchymal modulation was deemed necessary to induce FLR hypertrophy and minimize the risk of post-hepatectomy liver failure (PHLF). The choice of parenchymal modulation technique was guided by the following consideration: compared to portal vein ligation (PVL), “associating liver partition and portal vein ligation for staged hepatectomy” (ALPPS) would have been associated with higher morbidity and increased surgical workload due to the need for concomitant distal pancreatectomy with splenectomy, which would pose a greater risk of complications in the treatment of an otherwise slow-growing tumor. Therefore, we planned to perform right portal vein ligation along with left lobe metastasectomy and RFA, combined with pancreatic resection, during the first stage of the surgical intervention.

**Figure 3:**
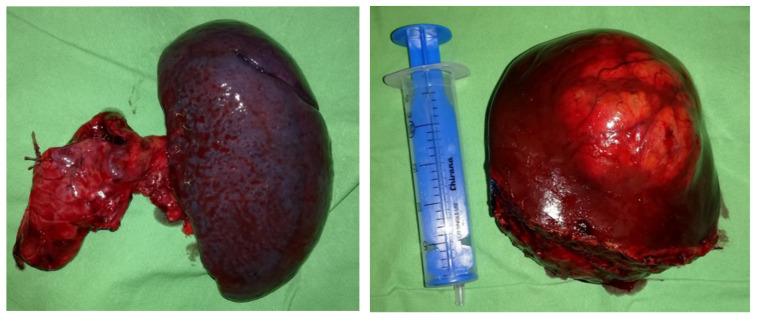
Surgical specimens. The spleen and pancreatic tail following resection (**left**), and a metastasis excised from liver segment III (**right**).

On 11 January 2021, the patient underwent distal pancreatic resection with splenectomy, along with metastasectomy from segment III of the left liver lobe (Figure 3) and the lower part of segment II. Histopathological analysis confirmed a primary grade 1 pancreatic neuroendocrine tumor (PanNET), along with grade 1 hepatic and lymph node metastases. The disease was staged as pT2 pN1 pM1, and complete (R0) resection was achieved for both the primary tumor and the hepatic metastases. Additionally, radiofrequency ablation (RFA) was performed on an intraparenchymal metastasis in segment II. To prepare for a planned right hemi-hepatectomy to address multiple metastases in the right liver lobe, a right portal vein ligation was also performed as parenchymal modulation.

### 2.3. Preoperative Planning and Second-Stage Surgery

After six weeks, a follow-up study was conducted to evaluate global and segmental liver function. Global liver function parameters remained stable despite parenchymal modulation, with a MELD score of 6, APRI score of 0.3, and CTP score of 5. However, ultrasound 2D-SWE revealed the development of mild fibrosis in the right lobe following portal vein ligation (PVL) (velocity: 1.53 m/s). No increase in elasticity indicative of fibrosis was observed in the hypertrophied left lobe (velocity: 1.3 m/s).

**Figure 4:**
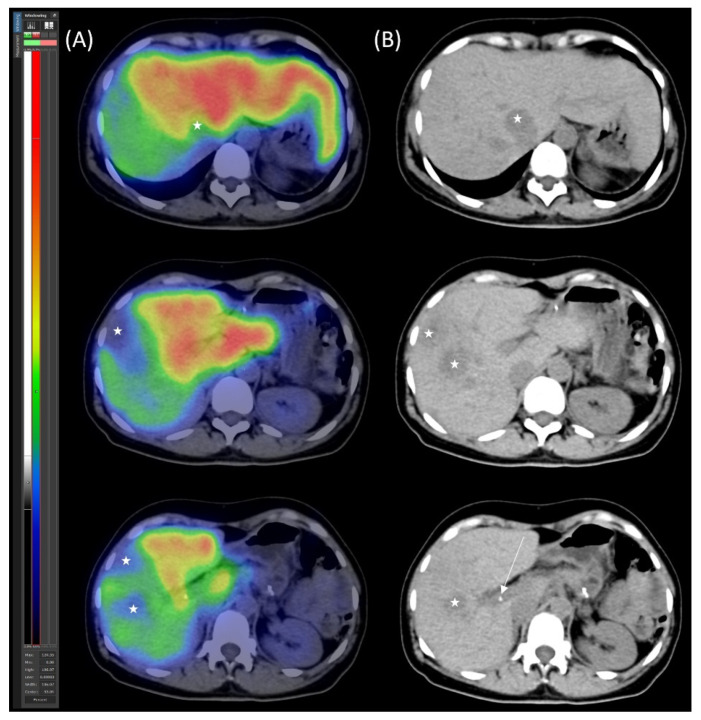
[^99m^Tc]Tc-mebrofenin SPECT/CT after parenchymal modulation. (**A**) Transaxial SPECT/CT and (**B**) native low-dose CT images show the distribution of functional hepatocytes, highlighting the dominance of the hypertrophied left lobe. The right lobe metastases appear as photopenic hypodense areas (marked with stars), with the arrow indicating the right portal vein ligation.

Control segmental liver function was assessed using [^99m^Tc]Tc-mebrofenin SPECT/CT (Figure 4). The imaging confirmed adequate hypertrophy of the future liver remnant (FLR), with the following values: FLR volume fraction (FLR-V%) at 46%, FLR functional volume fraction (FLR-FV%) at 66%, and FLR filtration rate at 3.16%/min/m^2^.

**Figure 5:**
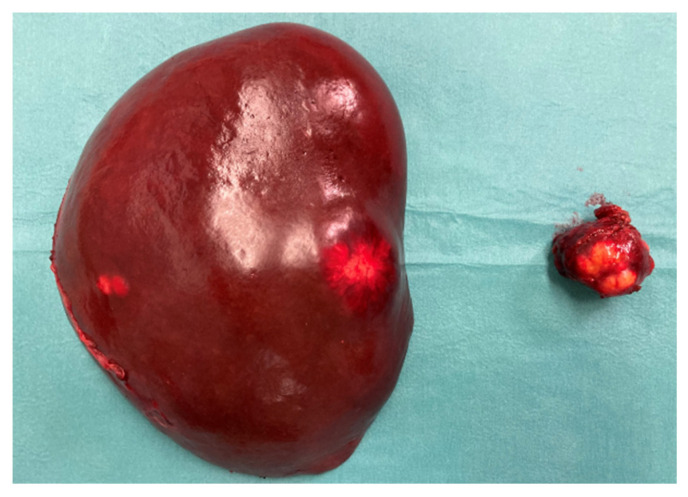
Surgical specimen. Resected right liver lobe with multiple metastatic lesions (**left**); metastasis from medial hepato-caval junction (**right**), consistent with PanNET metastases.

In March 2021, the patient underwent a successful right hemi-hepatectomy with metastasectomy from medial hepato-caval junction (Figure 5). Histopathological analysis confirmed multifocal metastatic neuroendocrine tumors originating from the previously resected pancreatic primary. However, the metastases showed an increased Ki-67 proliferation index of 6%, classifying these foci as grade 2 metastatic neuroendocrine tumors. Complete (R0) resection was achieved for all metastatic sites. No signs of clinically significant post-hepatectomy liver failure (PHLF) were observed in the postoperative period.

### 2.4. Follow-Up, Disease Progression, and Treatment

**Figure 6:**
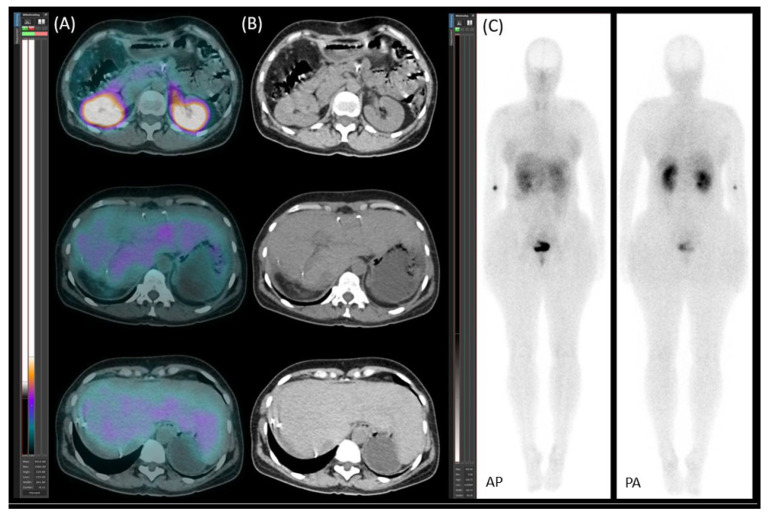
[^99m^Tc]Tc-EDDA/HYNIC-TOC scintigraphy with SPECT/CT. (**A**) Transaxial SPECT/CT, (**B**) transaxial CT, and (**C**) planar images (AP—anteroposterior; PA—posteroanterior) reveal no abnormal lesions.

A follow-up [^99m^Tc]Tc-EDDA/HYNIC-TOC somatostatin receptor scintigraphy with SPECT/CT (native, low-dose) was performed on 19 April 2021, revealing no somatostatin receptor-expressing lesions (Figure 6).

The multidisciplinary team (MDT) recommended ongoing follow-up to monitor the disease through serum chromogranin A (CgA) measurements and imaging studies.

On 29 July 2021, the patient’s serum CgA level was 52.5 ng/mL (reference range: 19.0–98.0), remaining within the normal range. A follow-up contrast-enhanced CT scan in November 2021 detected a solitary metastasis in the residual left liver lobe and an enlarged retroperitoneal lymph node.

**Figure 7:**
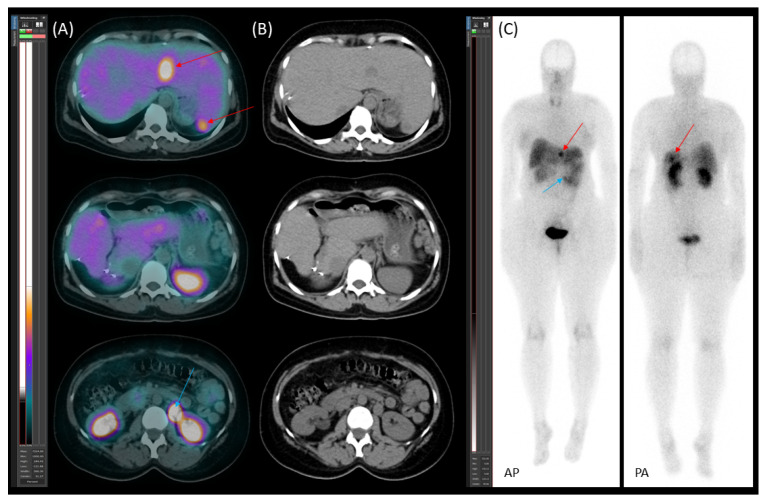
[^99m^Tc]Tc-EDDA/HYNIC-TOC scintigraphy with SPECT/CT. (**A**) Transaxial SPECT/CT, (**B**) transaxial CT, and (**C**) planar images (AP—anteroposterior; PA—posteroanterior) reveal new somatostatin receptor (SSTR)-positive lesions in the residual liver (red arrows) and para-aortic lymph node metastasis (blue arrow).

On 20 January 2022, [^99m^Tc]Tc-EDDA/HYNIC-TOC scintigraphy with SPECT/CT identified new somatostatin receptor-expressing lesions in the liver, as well as a positive lymph node along the abdominal aorta (Figure 7). These findings represented new disease progression compared to the previous scintigraphy on 19 April 2021. Due to recurrent disease, the patient was started on somatostatin analogue (SSA) therapy with lanreotide (120 mg, once a month). The patient tolerated SSA therapy well, without experiencing any significant side effects.

**Figure 8:**
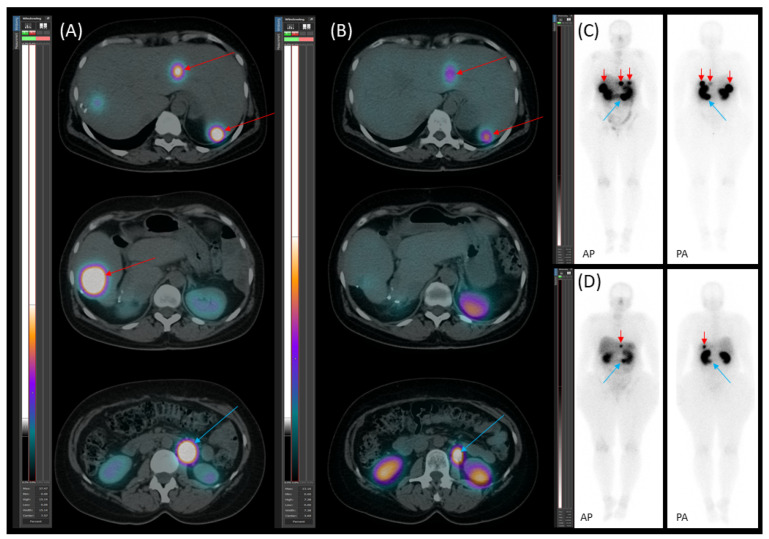
[^177^Lu]Lu-DOTA-TATE therapy response. Transaxial (**A**,**B**) SPECT/CT images and planar images (**C**,**D**) (AP—anteroposterior; PA—posteroanterior) obtained after the first (**A**,**C**) and fourth (**B**,**D**) therapy cycles with [^177^Lu]Lu-DOTA-TATE. The images demonstrate a reduction in both the number and activity of somatostatin receptor (SSTR)-positive lesions in the liver (red arrows), as well as a decrease in the size and activity of a para-aortic lymph node metastasis (blue arrow) following therapy.

Starting on 21 January 2023, the patient underwent four cycles of [^177^Lu]Lu-DOTA-TATE therapy due to disease progression despite treatment with somatostatin analogue (SSA). The therapy led to a reduction in both the number and metabolic activity of somatostatin receptor (SSTR)-positive lesions (Figure 8).

**Figure 9:**
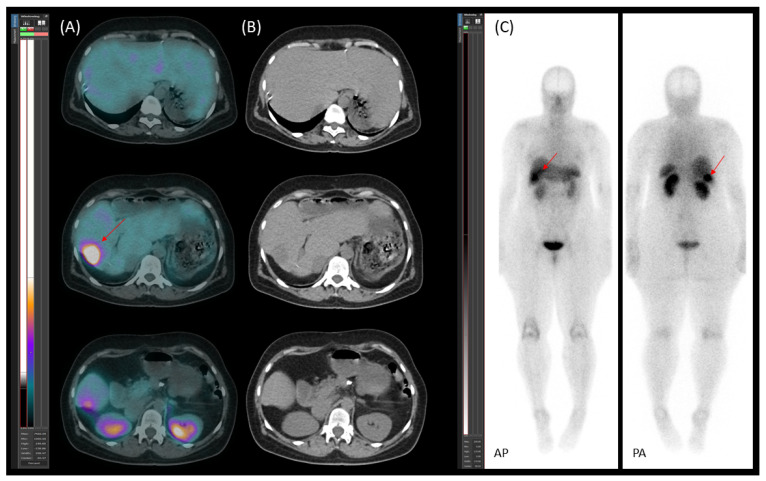
[^99m^Tc]Tc-EDDA/HYNIC-TOC scintigraphy with SPECT/CT. (**A**) Transaxial SPECT/CT, (**B**) transaxial CT, and (**C**) planar images (AP—anteroposterior; PA—posteroanterior) reveal a new somatostatin receptor (SSTR)-positive lesion (red arrow).

During follow-up, on 15 July 2024, [^99m^Tc]Tc-EDDA/HYNIC-TOC scintigraphy with SPECT/CT detected a new SSTR-positive liver metastasis at the site of a previously regressed lesion (Figure 9). However, the remaining SSTR-positive lesions exhibited decreased activity. In September 2024, an R0 resection was performed to remove the newly detected somatostatin receptor-expressing lesion in segment IV of the liver. Histology revealed that the neoplasm was a grade 3 metastatic neuroendocrine tumor with a Ki-67 index of 30%, indicating progression to a higher grade compared to both the original pancreatic primary and the previously resected liver metastases. 

The patient is currently in good general condition, with an ECOG (Eastern Cooperative Oncology Group) performance status of 0, indicating that she is fully active and asymptomatic, with 53 months having passed since the initial diagnosis of the disease.

### 2.5. Histopathological Findings

**Figure 10:**
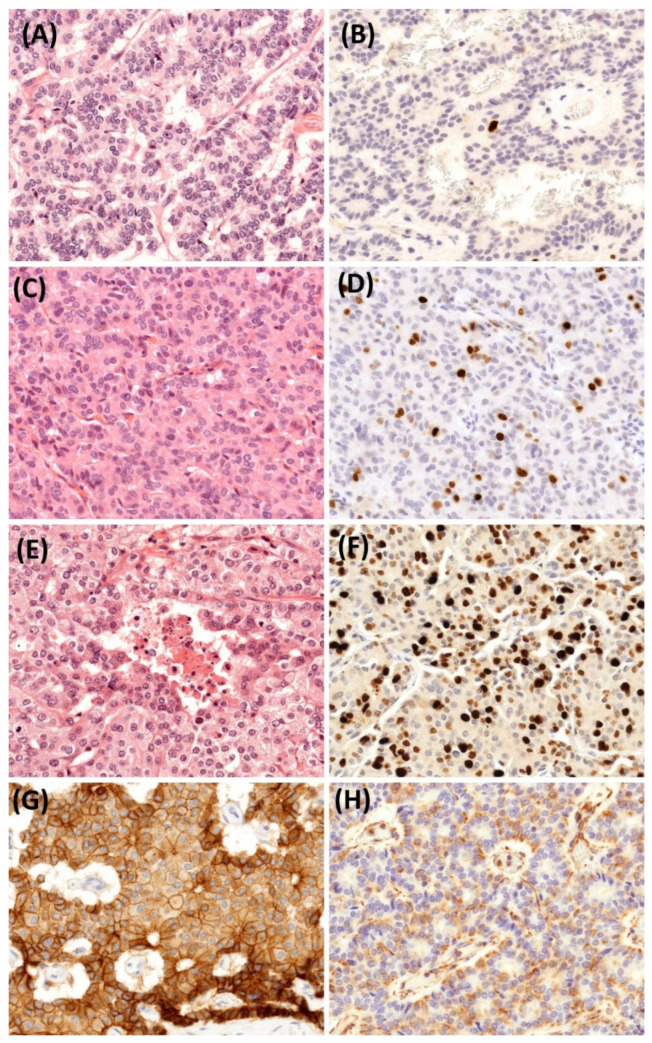
Representative images illustrate the dedifferentiation of the pancreatic neuroendocrine tumor (PanNET) from its primary site to metastatic lesions, highlighting the changes in tumor grade and the Ki-67 index. (**A**) shows the primary pancreatic NET, classified as grade 1 (Hematoxylin and Eosin [H&E], 400×), and (**B**) displays the corresponding Ki-67 index of 1% (400×). (**C**) depicts the liver metastasis from the hepato-caval junction, showing dedifferentiation to a grade 2 NET (H&E, 400×), and (**D**) shows the corresponding elevated Ki-67 index of 6% (400×). (**E**) depicts the last operated liver metastasis, which already dedifferentiated into a grade 3 NET, with small areas of necrosis, increased mitotic rate, and (**F**) a Ki-67 index of 30%. The tumor exhibited robust E-cadherin expression, with more than 90% of the cells displaying strong (2-3+) and complete circular membrane staining (**G**). Concurrently, vimentin expression was notably elevated, with moderate (2+) intensity observed in half of the tumor cells (**H**).

Histopathological examination of the resected primary pancreatic tumor revealed a well-differentiated neuroendocrine tumor (NET) exhibiting a nested and trabecular architecture. The tumor was composed of cells with minimal to moderate atypia, a low mitotic rate (1 mitosis per 2 mm^2^), a low Ki-67 index (1%), and no evidence of necrosis, consistent with the prior EUS-FNA diagnosis. Immunohistochemically, the tumor showed strong and diffuse expression of all commonly used neuroendocrine markers, including chromogranin A, synaptophysin, syntaxin-1, and insulinoma-associated protein 1 (INSM1). The concurrent liver metastases exhibited features identical to those of the primary tumor, including a similar Ki-67 index. However, a dedifferentiation process was observed in various liver metastases identified in subsequent liver resection samples. This was manifested by changes in mitotic activity, the Ki-67 index, and, consequently, tumor grade (Figure 10). Importantly, even the final grade 3 metastatic NET maintained an organoid nested and trabecular architecture, along with relatively bland cytomorphology and only a few small necrotic foci. These changes do not qualify the tumor as a neuroendocrine carcinoma (NEC). Immunohistochemical analysis of the grade 3 metastatic NET showed wild-type p53 expression, retained retinoblastoma (Rb) protein, no α-thalassemia/mental retardation syndrome X-linked (ATRX) loss, and strong expression of somatostatin receptor 2 (SSTR2) in 70% of the tumor cells. These findings effectively excluded the diagnosis of NEC. To elucidate why the primary tumor behaved aggressively despite being low-grade, we investigated markers associated with the epithelial–mesenchymal transition (EMT). Notably, the primary tumor co-expressed vimentin while unexpectedly retaining E-cadherin (Figure 10).

**Figure 11:**
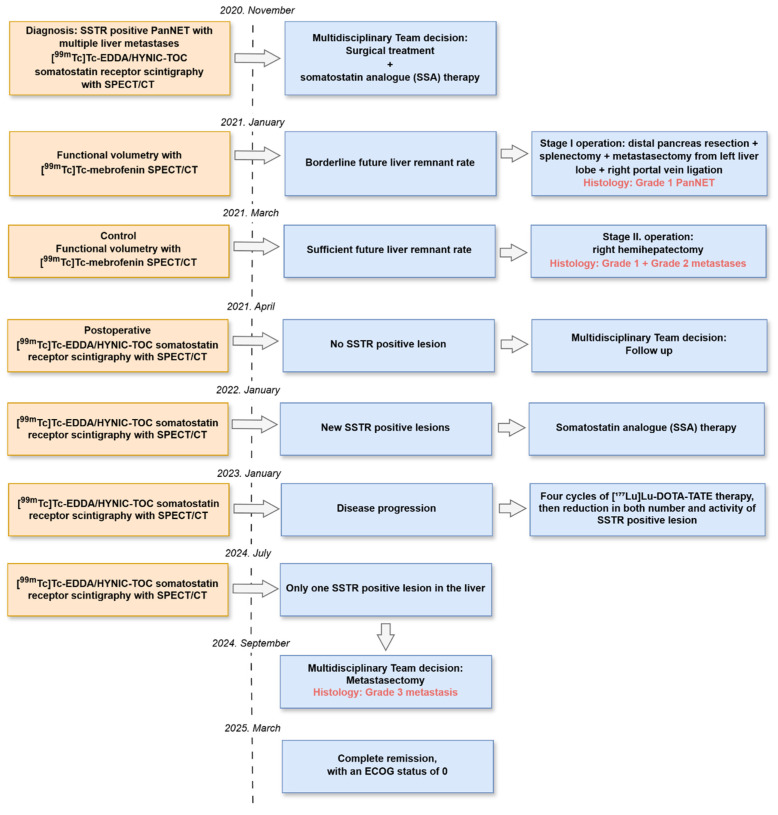
Case report timeline. SSTR: somatostatin receptor, PanNET: pancreatic neuroendocrine tumor, SPECT: single-photon emission computed tomography, CT: computed tomography, ECOG: Eastern Cooperative Oncology Group.

To provide a comprehensive overview, Figure 11 summarizes the key clinical events throughout the course of the case.

## 3. Discussion

### 3.1. Pathological Aspects and Tumor Grade Progression

Our patient presented with a pancreatic neuroendocrine tumor (PanNET) initially classified as grade 1, which, over time, progressed to grade 2 and subsequently to grade 3 liver metastases. The transition from a well-differentiated (grade 1) PanNET to a high-grade (grade 3) phenotype represents a rare phenomenon. In the literature, most cases of grade progression in gastroenteropancreatic neuroendocrine neoplasms (GEP-NENs) occur from grade 2 to grade 3. In a retrospective study, Grillo et al. reported grade progression between primary and metastatic sites, predominantly involving grade 2 tumors [15]. Raoul et al. described a case of a young woman with a long-standing grade 2 metastatic PanNET who experienced sudden disease progression after pregnancy, presenting with a liver metastasis histologically confirmed as a grade 3 large-cell neuroendocrine carcinoma [16]. One of the most common indicators of dedifferentiation is an increase in the Ki-67 proliferation index. Several studies have demonstrated that the Ki-67 index can vary over time and between the primary tumor and metastatic sites. This dynamic behavior suggests that tumor biology is not static but can evolve during disease progression and in response to therapy [15,17,18,19].

The mechanisms underlying dedifferentiation in pancreatic neuroendocrine tumors (PanNETs) are multifactorial and not fully elucidated. Intratumoral heterogeneity appears to play a central role, with distinct subpopulations of tumor cells exhibiting varying proliferative potential and differentiation status. Among these, poorly differentiated and more aggressive clones may initially exist in minor proportions but can expand over time, particularly under selective pressure from ongoing therapy. This dynamic clonal evolution may contribute to tumor progression [18,20].

Our patient received somatostatin analogue and peptide receptor radionuclide therapy (PRRT) during the disease course. There is also limited evidence suggesting that tumor progression may, in certain cases, be influenced by the neuroendocrine tumor-specific therapies themselves. Although somatostatin analogues (SSAs) typically achieve high rates of symptomatic control in the management of neuroendocrine tumors, the development of resistance over time has been reported, which may contribute to subsequent disease progression.

While PRRT is generally effective in well-differentiated SSTR-positive tumors, some studies suggest that treatment pressure may favor the expansion of more aggressive clones, potentially contributing to dedifferentiation in select cases [21,22].

In peptide receptor radionuclide therapy (PRRT), the somatostatin analogue is radiolabeled with lutetium-177, which targets NET cells by binding to the somatostatin receptor subtype 2 (SSTR2). During radioactive decay, Deoxyribonucleic Acid (DNA) damage is induced, leading to the death of NET cells. Heterogeneous SSTR2 expression within NET tumors can result in variable levels of DNA damage, influence the phenotype of recurrent tumors, and affect the therapeutic response, potentially through selection and expansion of more therapy-resistant cellular clones, as previously described by Feijtel et al. [23]. Further investigations are required to elucidate these mechanisms in greater detail.

In our case, despite the increased mitotic activity and elevated Ki-67 index, no evidence of transformation into NEC was observed. According to current data, neuroendocrine tumors (NETs) and NECs are pathogenetically and molecularly distinct entities. NECs typically present as primary high-grade tumors, with dedifferentiation driven by mutations in the P53 and Rb pathways, and these alterations were not detected in our case [24].

In our case, we analyzed the expression of E-cadherin and vimentin in the primary tumor as markers of the epithelial–mesenchymal transition (EMT). This dual expression could be a contributing factor to the metastatic behavior of PanNETs, despite their typically indolent nature. Interestingly, E-cadherin expression was preserved, while vimentin expression was high, suggesting an early phase of the EMT. Emerging evidence suggests that cancer stem-like cell populations, characterized by markers such as CD44 and Protein kinase D1 (PKD1), contribute to intratumoral heterogeneity and tumor maintenance in PanNETs. Guo et al. demonstrated that PKD1 signaling plays a pivotal role in sustaining cancer stem cells (CSCs), regulating gene expression profiles associated with the epithelial–mesenchymal transition (EMT), and promoting CSC self-renewal [25].

Through this mechanism, a subpopulation of cells in a partial EMT state may be maintained, supporting ongoing tumor progression, recurrence, and, potentially, dedifferentiation. Although these specific pathways were not investigated in our patient, they may represent contributing factors to the observed tumor evolution. Among the mechanisms implicated in dedifferentiation, epithelial–mesenchymal transition (EMT) may continue to be one of the key drivers of tumor dissemination and phenotypic transformation in PanNETs. Zhou et al. reported that in grade 1–2 PanNETs, high vimentin expression combined with reduced E-cadherin expression correlated with lymph node metastases, distant dissemination, and poor prognosis [26]. The examination of E-cadherin and vimentin expression in patients with grade 1–2 PanNETs holds potential as a prognostic marker for disease behavior and progression. However, further large-scale case–control studies are necessary to validate its clinical use. Taken together, our case highlights the complexity of PanNET progression and the likely interplay of multiple molecular mechanisms driving dedifferentiation. Continuous reassessment with both histopathological and functional imaging techniques remains critical for optimal disease management.

### 3.2. Somatostatin Receptor Imaging and Clinical Relevance

In our case, despite the increase in tumor grade within the metastatic sites, the lesions continued to express somatostatin receptors (SSTRs). No significant discordance was observed between imaging findings and the biological behavior of the tumor during disease progression. As the pancreatic grade 1 neuroendocrine tumor progressed to grade 3 metastatic disease, somatostatin receptor expression was consistently maintained and detectable on [^99m^Tc]Tc-EDDA/HYNIC-TOC SPECT/CT. This finding underscores the ongoing utility of SSTR-based imaging, even in higher-grade lesions. Furthermore, the preserved SSTR expression provided the basis for initiating peptide receptor radionuclide therapy (PRRT), which contributed to disease stabilization at this advanced stage. Although [^68^Ga]Ga-DOTATATE PET/CT is currently regarded as the gold standard for imaging somatostatin receptor (SSTR)-positive neuroendocrine tumors due to its superior sensitivity and spatial resolution, its routine availability remains limited in many centers. In our case, [^99m^Tc]Tc-EDDA/HYNIC-TOC SPECT/CT was employed, as PET/CT was not available at the time of diagnosis in our institution. According to the 2020 ESMO Clinical Practice Guidelines, SSTR scintigraphy remains an acceptable alternative when PET/CT is not accessible, particularly when combined with hybrid SPECT/CT to improve anatomical localization. Therefore, our diagnostic approach was fully consistent with current European recommendations and provided adequate accuracy for staging, treatment planning, and therapeutic decision-making [5].

### 3.3. Therapeutic Considerations

#### 3.3.1. Surgical Treatment

Surgery is the treatment of choice for local or locoregional disease in PanNET grade 1 and grade 2. The surgical approach is also indicated in selected patients with stage IV PanNETs who present with exclusive or predominant liver involvement after careful evaluation of tumor grading, distribution of liver metastases, and primary tumor localization. In combination with surgical resection, ablative locoregional treatments are also valid options for the management of liver metastases. The choice of procedures depends on local expertise, the extent and localization of liver metastases, and the degree of liver involvement [5].

#### 3.3.2. Preoperative Planning

In our case, a multistage liver resection strategy was implemented with meticulous preoperative planning to prevent post-hepatectomy liver failure (PHLF), a complication associated with high mortality following major liver resections. PHLF remains one of the most common causes of major morbidity, with reported incidence rates ranging from 9% to 30% after extended liver resections, and continues to represent a key determinant of postoperative mortality. As the initial step in preoperative planning, the patient’s global liver function was assessed using clinical scoring systems based on laboratory parameters, in accordance with the recommendations of the E-AHPBA–ESSO–ESSR Innsbruck consensus guidelines on preoperative liver function assessment prior to hepatectomy [8,27].

The indocyanine green test may provide a more accurate assessment of global liver function, as it reflects hepatocellular clearance [28]; however, it does not provide information about segmental liver function. Since the patient developed an allergic reaction following intravenous administration of ICG, the procedure could not be performed. In our case, the fibrotic process before and following parenchymal modulation was assessed non-invasively using ultrasound two-dimensional shear wave elastography (2D-SWE). Parenchymal modulation induces atrophy in the resectable portion of the liver while stimulating a compensatory hypertrophic response in the contralateral lobe [29]. In our patient, fibrosis was present in the atrophic liver lobe, while the hypertrophic lobe, corresponding to the future liver remnant (FLR), showed no evidence of fibrosis. Indeed, 2D-SWE provides valuable insight into liver tissue characteristics, measuring liver elasticity to assess the degree of fibrosis [30]. However, while ultrasound 2D-SWE is effective in detecting liver fibrosis, it does not provide volumetric data, which is essential for comprehensive preoperative planning. CT-based volumetry is a well-established tool for assessing the future liver remnant volume, and most centers recommend an FLR volume of at least 25% for patients with normal liver parenchyma undergoing major hepatectomy [12]. Nevertheless, CT volumetry provides no information about the regional functional capacity of hepatocytes. In this context, functional assessment with [^99m^Tc]Tc-mebrofenin SPECT/CT has emerged as a reliable method for predicting PHLF, offering simultaneous quantification of both total and segmental liver function. Functional volumetry allows for the calculation of not only the FLR volume but also FLR filtration, which can better identify patients at risk of PHLF [8,11]. Bakos et al. demonstrated that FLR filtration determined by dynamic [^99m^Tc]Tc-mebrofenin SPECT/CT was a significant predictor of clinically relevant post-hepatectomy liver failure [10]. During our surgical planning, the initial functional volumetric assessment indicated that the FLR proportion was sufficient to proceed with resection; however, the FLR filtration was below the literature-defined cutoff value [11], which significantly influenced the surgical decision-making process and led to the choice of parenchymal modulation in order to enhance patient safety. Our patient did not develop clinically relevant PHLF [31] after two-stage major liver resection, demonstrating that preoperative functional volumetry is a valuable tool for guiding objective decisions regarding parenchymal modulation. We recommend the broader use of [^99m^Tc]Tc-mebrofenin SPECT/CT functional volumetry in clinical practice, as it facilitates the identification of patients at risk of PHLF and aids in optimizing surgical outcomes. This approach has the potential to improve safety and decision-making in complex liver resections.

#### 3.3.3. Parenchymal Modulation Technique

In the selection of the parenchymal modulation techniques, we applied portal vein ligation, which has a lower perioperative risk compared to Associating Liver Partition and Portal vein ligation for Staged hepatectomy (ALPPS). We considered this particularly important in our case, where we also performed distal pancreatectomy with splenectomy during the first stage of surgical intervention. Although the FLR increase rate is higher in the case of ALPPS, we found it safer to use portal vein ligation in our case for the reasons mentioned above [32,33,34].

#### 3.3.4. Neuroendocrine Tumor-Specific Therapies

The use of adjuvant somatostatin analogue (SSA) therapy following resection of pancreatic neuroendocrine tumors (PanNETs) remains a matter of ongoing debate. According to the current ESMO clinical practice guidelines, there are no prospective, randomized clinical trials supporting the routine use of adjuvant therapy in patients with grade 1/grade 2 NETs; therefore, it is generally not recommended [5]. However, recent retrospective studies have identified several high-risk factors, including PanNET G3 grade, pancreatic duct dilatation, and perineural invasion, which serve as independent predictors of early recurrence following curative resection. These risk factors have been incorporated into novel prognostic models that demonstrate superior predictive accuracy compared to traditional tumor-node-metastasis (TNM) staging systems. Importantly, in these high-risk patient subgroups, adjuvant long-acting SSA therapy was associated with significantly reduced recurrence rates and improved overall survival [35,36,37,38].

Consequently, the use of adjuvant SSA treatment may warrant careful, individualized consideration depending on the patient’s risk profile, although further evidence is needed to define its role more precisely.

For patients with progressive well-differentiated NETs under standard SSA therapy, the CLARINET FORTE phase 2 trial demonstrated that dose intensification, by reducing the administration interval to 14 days, may prolong progression-free survival, particularly in patients with Ki-67 ≤ 10%, while maintaining safety and quality of life, thus offering a potential therapeutic alternative before escalation to more aggressive or less tolerable treatments [39]. Prior to the initiation of the CLARINET FORTE phase 2 trial, only a few retrospective or small prospective studies had assessed response rates to escalated-dose somatostatin analogues in patients with gastroenteropancreatic neuroendocrine tumors (GEP-NETs). This topic was also addressed in the systematic review by Chan et al. [40].

Peptide receptor radionuclide therapy (PRRT) is a recommended treatment option for patients with advanced, somatostatin receptor-positive neuroendocrine tumors (NETs), particularly in patients who progress on somatostatin analogue (SSA) therapy and meet the general eligibility criteria for PRRT [5]. The NETTER-1 trial demonstrated that PRRT significantly prolongs progression-free survival (PFS) and improves quality of life in this patient population [41].

In our case, tumor progression was characterized by transition from grade 1 PanNET to higher-grade liver metastases over time, with eventual development of grade 3 lesions following PRRT. This phenomenon underscores the potential for PanNETs to evolve into higher-grade disease. A major step forward in this field is the use of first-line [^177^Lu]Lu-DOTA-TATE in patients with higher-grade (grade 2–3), well-differentiated, advanced gastroenteropancreatic NETs, as evaluated in the NETTER-2 phase III trial. The study demonstrated that first-line [^177^Lu]Lu-DOTA-TATE combined with octreotide long-acting repeatable (LAR) significantly prolonged progression-free survival in patients with advanced grade 2 or 3 gastroenteropancreatic neuroendocrine tumors [42]. These novel findings open up new possibilities for the use of therapeutic isotopes in the management of higher-grade tumors.

#### 3.3.5. Disease Monitoring

The patient was monitored postoperatively with chromogranin A (CgA) measurements and conventional contrast-enhanced CT (ceCT) scans. CgA levels remained within the normal range after surgery. However, a new hepatic nodule was identified on ceCT, and somatostatin receptor scintigraphy confirmed SSTR expression, suggesting recurrent disease. CgA is a widely used biomarker for NETs, as its levels can correlate with tumor burden, differentiation, and progression. However, CgA levels alone are not always reliable, as small or well-differentiated tumors may not produce elevated levels [43]. Therefore, when interpreting CgA levels, monitoring trends over time is more informative than relying solely on whether values exceed a predefined cutoff threshold [44]. This trend-based approach is essential for assessing disease progression and recurrence with greater accuracy.

## 4. Limitation

The patient’s follow-up was disrupted due to the COVID-19 pandemic, resulting in missed medical examinations between April 2021 and November 2021.

## 5. Conclusions

This case report presents a clinically interesting and technically detailed case of a grade 1 pancreatic neuroendocrine tumor (PanNET) with hepatic metastases, which, over time, progressed to grade 3 disease. Our patient underwent comprehensive multidisciplinary treatment, resulting in excellent clinical outcomes. This case illustrates the multifaceted role of functional imaging in the management of pancreatic neuroendocrine tumors, encompassing preoperative planning, liver function assessment, and disease monitoring. The integration of advanced nuclear medicine techniques, such as [^99m^Tc]Tc-mebrofenin-based SPECT/CT and somatostatin receptor imaging, facilitated personalized and timely therapeutic decisions throughout the different stages of the disease course. The transition from low-grade to high-grade disease emphasizes the importance of ongoing surveillance and a dynamic multidisciplinary approach. Further research is warranted to better elucidate the biological mechanisms underlying tumor grade progression and to refine the role of targeted therapies in advanced PanNET management.

## Data Availability

The raw data supporting the conclusions of this article will be made available by the authors without undue reservation.
